# A survey-based study: assessing inpatient attending perspectives on teaching learners, feeling valued, and symptoms of burnout

**DOI:** 10.1186/s12909-024-05757-9

**Published:** 2024-07-29

**Authors:** William C. Lippert, Jessica L. McCutcheon, Gregory B. Russell, Kenneth J. Singhel, Christina M. Rinaldi, Suma Menon, Parag A. Chevli, Jacqueline D. Lippert, Edward H. Ip, Chi-Cheng Huang

**Affiliations:** 1 Department of Internal Medicine, Section on Hospital Medicine, Wake Forest University School of Medicine, Atrium Health Wake Forest Baptist, 1 Medical Center Boulevard, Winston-Salem, NC 27157 USA; 2 Department of Internal Medicine, Wake Forest University School of Medicine, Atrium Health Carolinas Medical Center, 1000 Blythe Boulevard, Charlotte, NC 28203 USA; 3 Department of Biostatistics and Data Science, Wake Forest University School of Medicine, 525 Vine Street, Winston-Salem, NC 27157 USA; 4 Department of Internal Medicine, Section on General Internal Medicine, Wake Forest University School of Medicine, Atrium Health Wake Forest Baptist, 1 Medical Center Boulevard, Winston-Salem, NC 27157 USA

**Keywords:** Physician wellbeing, Physician satisfaction, Physician fulfillment, Physician wellness, Inpatient teaching

## Abstract

**Background:**

Physician burnout is rising, especially among academic physicians facing pressures to increase their clinical workload, lead administrative tasks and committees, and be active in research. There is a concern this could have downstream effects on learners’ experiences and academic physician’s ability to teach learners on the team.

**Methods:**

A 29-question RedCap survey was electronically distributed to 54 attending physicians within an academic learning health system who oversaw the General Medicine inpatient teaching services during the 2022–2023 academic year. The aims were to assess this cohort of attending physicians’ experiences, attitudes, and perceptions on their ability to effectively teach learners on the team, feeling valued, contributors to work-life balance and symptoms of burnout, Fisher’s Exact Tests were used for data analysis.

**Results:**

Response rate was 56%. Attendings splitting time 50% inpatient / 50% outpatient felt that team size and type of admissions model affected their ability to effectively teach learners (*p* = 0.022 and *p* = 0.049). Attendings with protected administrative time felt that non-patient care obligations affected their ability to effectively teach the learners (*p* = 0.019). Male attendings and attendings with ≤ 5 years of General Medicine inpatient teaching experience felt less valued by residency leadership (*p* = 0.019 and *p* = 0.026). 80% of attendings experienced emotional exhaustion, and those with > 10 weeks on a General Medicine inpatient teaching service were more likely to experience emotional exhaustion (*p* = 0.041). Attendings with > 10 weeks on a General Medicine inpatient teaching service and those who were a primary caregiver were more likely to experience depersonalization (*p* = 0.012 and *p* = 0.031). 57% of attendings had reduced personal achievement.

**Conclusions:**

Institutions should seek an individual and organizational approach to professional fulfillment. Special attention to these certain groups is warranted to understand how they can be better supported. Further research, such as with focus groups, is needed to address these challenges.

## Background

Over the last few decades, there is an understanding that the United States (US) healthcare system will need to make dramatic changes to support the increasing demands of our profession, including the academic teaching mission. It is projected that by 2034 the US population will increase by 10.6%, and by 42.4% for patients over the age of 65 years old. Unfortunately, this increase in demand for healthcare is not met by the supply of physicians in the US with a predicted shortage of 17,800 primary care physicians and 21,000 subspecialty physicians by 2034 [1].

The etiology of this physician shortage is multifactorial with burnout proving to be a major contributing factor. For example, US studies note a prevalence of physician burnout symptoms that exceed 50% [2, 3]. More recent studies during the COVID-19 pandemic estimate the numbers to be even higher, and that 1 in 5 physicians intend to leave the profession altogether [4]. This is an increase from the 6–7% per year that was reported prior to the pandemic based on data from the Association of American Medical Colleges (AAMC) [5]. Burnout also has substantial economic impacts on health care organizations with the cost to replace a physician ranging from $500,000 to $1,000,000, and this is without considering the impact of physician turnover on our patients [3].

Moreover, academic physicians continue to experience pressures to increase clinical workload, lead administrative tasks and committees, and bear the financial and time constraints of research [6, 7]. This is all the while upholding a mission to provide high-quality teaching and attempting to lead by example for our learners and future physicians [8–10]. To fulfill the AAMC vision of teaching the next generations of clinicians, academic physicians must feel a sense of professional satisfaction, fulfillment, and wellbeing to complete their tasks at hand. Given the need to properly identify factors that contribute to or diminish professional fulfillment and address the barriers that exist at the personal and institutional levels, we aimed to investigate inpatient attending physician’s demographics, perceptions of teaching learners on the team, feeling valued, and symptoms that could suggest burnout across the two –major inpatient teaching sites for Wake Forest University School of Medicine.

## Methods

To comprehensively assess inpatient attending physician experiences, attitudes, and perceptions on the following:

- Ability to effectively teach learners on the team
- Their feelings of value and work-life balance
- Their symptoms of burnout

Therefore, we employed a rigorous methodological approach that is detailed below.

### Participant selection

The study targeted attending physicians who supervised the General Medicine inpatient teaching services during the 2022–2023 academic year. The research focused on two prominent academic health centers: (1) Atrium Health Wake Forest Baptist (AHWFB), which includes Atrium Health Wake Forest Baptist Medical Center and Atrium Health High Point Medical Center, and (2) Atrium Health Carolinas Medical Center (AHCMC). These centers were strategically chosen within the Advocate Health Southeast Region to ensure diversity in experiences across different academic settings. 54 attending physicians at these centers met the inclusion study criteria and were invited to participate. Their responses formed the basis for this study.

### General medicine inpatient teaching services

The structure of the General Medicine Inpatient Teaching Services varied at each site.

For AHWFB, the general structure on any given day is 1 supervising attending, 1 upper-level resident, 2–3 interns, and 1–4 medical students. The team cap is 16 patients, and a drip system is used for new patients to the team, meaning that the team can receive a new patient (e.g., admission or transfer) at any point in time if the census drops below their team cap. The attending is typically on-service for 7 consecutive days.

For AHCMC, the general structure on any given day is 1 supervising attending, 1 upper-level resident, 2–3 interns, and 1–3 medical students. The team cap is 18 patients, and a bolus system is used for new patients to the team, meaning that they only accept new patients (e.g., admissions or transfers) to the team on their “call” day, which is every 48 h. The attending is typically on-service for 14 consecutive days.

### Survey development

The survey instrument underwent an iterative development process, including:

- collaboration with expert medical educators from the AHCMC and AHWFB campuses, who are authors on the current study
- experts in survey design and statistical analysis from the Wake Forest University School of Medicine Clinical & Translational Science Institute (CTSI)
- a group of external medical educators from outside academic institutions who provided direct feedback on the survey during its development

The final instrument, presented in Appendix, Fig. 1, consisted of 29 carefully crafted questions. These questions were designed to capture a wide range of factors influencing attending physician experiences, demographic data, perceptions and attitudes on their ability to teach learners, feeling valued, job satisfaction, and symptoms of burnout who supervised the General Medicine inpatient teaching services.

### Survey deployment

We utilized Research Electronic Data Capture (REDCap; Nashville, TN, USA), a secure web application for building and managing online surveys, to deploy the survey. E-mail invitations containing a link to the survey (Appendix, Fig. 2) were sent to eligible participants starting on July 1, 2023. To enhance participation rates, two automated reminders were dispatched bi-weekly to those who had not yet completed the survey. Additionally, the study was announced during local staff meetings at AHWFB and AHCMC to reinforce awareness and encourage participation. The survey period ended on August 12, 2023.

### Ethical considerations

Prior to survey dissemination, ethical approval was obtained from the Institutional Review Board to ensure the protection of participants’ rights and confidentiality. The study adhered to ethical guidelines, and all responses were collected anonymously. A neutral third-party investigator, uninvolved in determining attending physician roles or remuneration, was appointed to analyze the responses, further safeguarding participant confidentiality.

### Data analysis

Quantitative data collected through the survey were subjected to robust statistical analyses. We examined attendings perception of being able to effectively teach, feeling valued, and symptoms of burnout with demographic information and personal/professional traits outlined in Table 1 by using Fisher’s Exact Tests. P-values < 0.05 were assumed to be statistically significant and SAS (Version 9.4; Cary, NC, USA) was used for all analyses.

**Table 1:** Inpatient attending demographic information based on each site

Respondent Demographics	Response	Atrium Health Wake Forest Baptist (AHWFB) (*n* = 19), n (%)	Atrium Health Carolinas Medical Center (AHCMC) (*n* = 11), n (%)	*P*- value
Gender	Female	10 (53)	6 (55)	>0.99
	Male	8 (42)	4 (36)	
	Prefer Not to Say	1 (5)	1 (9)	
Race/Ethnicity	Asian or Pacific Islander	6 (32)	1 (9)	0.35
	Black or African American	1 (5)	0 (0)	
	White or Caucasian	7 (37)	7 (64)	
	Race/ethnicity not listed	2 (11)	0 (0)	
	Prefer Not to Say	3 (16)	3 (27)	
Primary caregiver	Yes	10 (53)	5 (45)	>0.99
	No	9 (47)	6 (55)	
Years in Practice	0–2 years	2 (11)	1 (9)	0.71
	2–5 years	5 (26)	2 (18)	
	6–10 years	4 (21)	1 (27)	
	11–15 years	4 (21)	2 (18)	
	15–20 years	3 (16)	2 (18)	
	> 20 years	1 (5)	3 (27)	
Years at current institution on an inpatient teaching service	0–2 years	4 (21)	1 (9)	0.084
	2–5 years	5 (26)	3 (27)	
	6–10 years	9 (47)	2 (18)	
	11–15 years	1 (5)	3 (27)	
	15–20 years	0 (0)	1 (9)	
	> 20 years	0 (0)	1 (9)	
Weeks on inpatient teaching service	< 2 weeks	0 (0)	0 (0)	*0.024
	2–4 weeks	2 (11)	0 (0)	
	4–6 weeks	4 (21)	0 (0)	
	6–8 weeks	4 (21)	1 (9)	
	8–10 weeks	5 (26)	1 (9)	
	> 10 weeks	4 (21)	9 (82)	
Setting spend > 50% of your clinical time	Inpatient	12 (63)	10 (91)	0.33
	Outpatient	2 (11)	0 (0)	
	Even Split 50% Inpatient, 50% Outpatient	5 (26)	1 (9)	
Faculty with any protected time	Yes	17 (89)	9 (81)	0.61
	No	2 (11)	2 (18)	

*Denotes statistical significance with *p* < 0.05

The survey’s last question was an open-ended question asking the following “Feel free to share any other comments or suggestions on improving your experience while serving as a supervising attending for the General Medicine Inpatient Teaching Services” (Appendix, Fig. 1). The authors reviewed all answers to this question by the survey respondents (n = 12), and selective comments that provided additional context, clarification, or elaboration to some of the questions asked in the survey were included in the Results section.

## Results

A total of 30 attending physicians completed the survey (response rate: 56%). 63% of the attendings worked at AHWFB (19 attendings; response rate at this site: 56%), and 37% at AHCMC (11 attendings; response rate at this site: 55%). Additional attending respondent demographic data can be found in Table 1. There was a significant difference between AHWFB and AHCMC in the number of weeks spent on General Medicine inpatient teaching services (*p* = 0.024). AHCMC attending physicians had significantly more weeks, otherwise no other differences were noted (Table 1).

### Ability to effectively teach learners on the team

Overall, all 30 attending respondents reported that they were either “very satisfied” or “satisfied” with their overall experience supervising the General Medicine inpatient teaching services. However, those attendings who worked 50% inpatient and 50% outpatient felt that the number of learners on the team and the type of admissions model (e.g., bolus or drip system) affected their ability to effectively teach the learners on the team in comparison to those attendings who worked the majority of their time (> 50%) in the inpatient or outpatient settings (*p* = 0.022 and *p* = 0.049, respectively). One attending expressed, “the teams are often oversaturated with learners,” and another stated that with the drip admissions system, “I never really get to do chalk-talks, which is a bummer. The residents always seemingly are too busy.” Those attendings with administrative protected time as part of their full-time equivalent (FTE) felt that non-patient care obligations (e.g. administrative meetings; research) affected their ability to effectively teach the learners on the team in comparison to those attendings who did not (*p* = 0.019). Other factors including patient census, attending length of time on General Medicine inpatient teaching service (e.g., working 7-days consecutively, or 14-days consecutively), and current system initiatives/quality metrics (e.g. prioritizing early discharges) had no significant effect on an attendings’ ability to effectively teach the learners on the team. However, those attendings who had *≤* 5 years of experience in practice had a trend towards significance in their ability to effectively teach the learners on the team and to balance current system initiatives/quality metrics (*p* = 0.086). One attending wrote that the quality metrics “has caused unnecessary stress/anxiety among the team members… and have completely changed the way I have rounded as a result of these system efforts.” Another attending reported that “it always feels like “Big Brother” is looking over our shoulders and judging how we’re taking care of our patients.” Table 2 outlines some of the significant findings.

**Table 2:** Survey questions assessing inpatient attending ability to effectively teach residents and students based on their demographic information. Only those questions and demographic information that were statistically significant or trending towards significance based on a p-value < 0.05 are included. *indicates statistical significance with a p-value < 0.05

Question	Number of years practice (split at *≤* 5 years)	Number of years at current institution (split at *≤* 5 years)	Number of years at current institution (split at *≤* 10 years)	Setting (e.g. 50% inpatient/50% outpatient (Both) vs. primarily inpatient or outpatient)	Number of inpatient clinical weeks (split at *≤* 10 weeks)	Academic protected time (< 100% vs. 100%)
Number of residents and medical students on a team affected ability to effectively teach learners on the team	0.06 *≤* 5 years, 30% > 5 years, 0%	0.36 *≤* 5 years, 23% > 5 years, 0%	0.25 *< 10* years, 13% > 10 years, 0%	0.022* Both, 0% Inpatient or outpatient, 13%	0.94 *< 10* weeks, 12% > 10 weeks, 8%	0.93 *< 100%*, 0% 100%, 13%
Patient admission model affected ability to effectively teach learners on the team	0.32 *≤* 5 years, 60% > 5 years, 25%	0.40 *≤* 5 years, 54% > 5 years, 24%	0.49 *< 10* years, 38% > 10 years, 33%	0.049* Both, 0% Inpatient or outpatient, 46%	0.61 *< 10* weeks, 29% > 10 weeks, 46%	0.092 *< 100%*, 17% 100%, 39%
Non-patient care obligations affected ability to effectively teach learners on the team	0.12 *≤* 5 years, 50% > 5 years, 30%	0.067 *≤* 5 years, 46% > 5 years, 29%	0.91 *< 10* years, 38% > 10 years, 33%	0.73 Both, 17% Inpatient or outpatient, 42%	0.51 *< 10* weeks, 35% > 10 weeks, 38%	0.019*^ *< 100%*, 33% 100%, 35%
Current system initiatives/quality metrics affected ability to effectively teach learners on the team	0.054 *≤* 5 years, 60% > 5 years, 15%	0.086 *≤* 5 years, 46% > 5 years, 18%	0.99 *< 10* years, 29% > 10 years, 33%	0.75 Both, 17% Inpatient or outpatient, 33%	0.35 *< 10* weeks, 29% > 10 weeks, 31%	0.89 *< 100%*, 17% 100%, 35%

**%** of respondents who chose “Always” or “Often” shown in table* indicates statistically significant difference

^ 67% of those with < 100% chose “Rarely/Never” versus 22% of those with 100% funding

### Perceptions of feeling valued

In terms of attendings assessment of feeling valued by hospital leadership, there were no statistically significant findings observed. However, one attending affirmed that they “do not feel valued whatsoever by hospital leadership” and another said that their “value as CLINICIAN EDUCATORS has become devalued”. There was a trend towards significance in attendings who are the primary caregiver for either a family member or friend and having feelings of being less valued by hospital leadership than those who were not (*p* = 0.098). In terms of attendings assessment of feeling valued by internal medicine residency leadership, male attendings felt less valued in comparison to female attendings (*p* = 0.019). Further, those attendings with *≤* 5 years of experience supervising a General Medicine inpatient teaching service felt less valued than those attendings with > 5 years of experience (*p* = 0.026). One attending stated that “I do think the IM residency leadership values me as an attending, but I do not think others share this same sentiment. I think the IM residency leadership should do more things to thank us for supervising the residents and students.” In terms of attendings feeling valued by the residents and medical students, there were no observed statistical differences noted, and no specific comments about this in the qualitative question that was asked. Table 3 outlines some of the significant findings.

### Symptoms of burnout

Twenty-four attendings (80%) had feelings either “often” or “sometimes” of being emotionally exhausted due to their work. More specifically, those attendings who had > 10 weeks on a General Medicine inpatient teaching service had more feelings of emotional exhaustion than those with *≤* 10 weeks (*p* = 0.041). Also, when compared by site, attendings primarily working at AHWFB with > 10 weeks on a General Medicine inpatient teaching service were more likely to report symptoms of emotional exhaustion than those attendings at AHCMC with > 10 weeks (*p* = 0.049). Those attendings with *≤* 10 years of experience at their current institution were also more likely to report having feelings of emotional exhaustion than those with > 10 years (*p* = 0.041). Those attendings with administrative protected time as part of their full-time equivalent (FTE) had a trend towards significance in having feelings of emotional exhaustion (*p* = 0.054). Table 3 outlines some of the significant findings.

Eleven attendings (37%) experienced feelings of depersonalization either “often” or “sometimes.” More specifically, those attendings who had > 10 weeks on a General Medicine inpatient teaching service had more feelings of depersonalization than those with *≤* 10 weeks (p = 0.012). When compared by site, attendings from AHWFB with more than > 10 weeks on a General Medicine inpatient teaching service were more likely to report symptoms of depersonalization than attendings at AHCMC with more than > 10 weeks (p = 0.023). Furthermore, those attendings who were the primary caregiver to a family member or friend were more likely to experience feelings of depersonalization (p = 0.031). Table 3 outlines some of the significant findings.

Seventeen of the attendings (57%) reported either “often” or “sometimes” having feelings of reduced personal achievement. When compared by site, attendings from AHWFB with more than > 10 weeks on a General Medicine inpatient teaching service had more feelings of reduced personal achievement than attendings from AHCMC with more than > 10 weeks on a General Medicine inpatient teaching service, but this was not statistically significant (*p* = 0.079). There were no other observed statistical differences noted regarding emotional exhaustion, depersonalization or reduced personal achievement. Table 3 outlines some of the significant findings.

**Table 3:** Survey questions assessing inpatient attending feelings of being valued and burnout questions based on their demographic information. Only those questions and demographic information that were statistically significant or trending towards significance based on a p-value < 0.05 are included. *indicates statistical significance with a p-value < 0.05

Question	Gender (Female, F; Male, M)	Primary caregiver (CG)	Number of years at current institution (split at *≤* 5 years)	Number of years at current institution (split at *≤* 10 years)	Number of inpatient clinical weeks (split at *≤* 10 weeks)	Academic protected time
Feel valued by hospital leadership	0.22 F, 38% M, 17%	0.098 CG, 13% Non-CG,40%	0.88 *≤* 5 years, 23% > 5 years, 29%	0.19 *< 10* years, 29% > 10 years, 17%	0.88 *≤* 10 weeks, 29% > 10 weeks, 23%	0.24 *< 100%*, 33% 100%, 22%
Feel valued by internal medicine residency leadership	0.019* F, 69% M, 42%	0.99 CG: 53% Non-CG, 60%	0.026* *≤* 5 years, 38% > 5 years, 71%	0.20 *< 10* years, 46% > 10 years, 100%	0.22 *< 10* weeks, 59% > 10 weeks, 54%	0.48 *< 100%*, 50% 100%, 57%
Feelings of emotional exhaustion	0.54 F, 44% M, 33%	0.73 CG: 47% Non-CG,33%	0.56 *≤* 5 years, 31% > 5 years, 47%	0.041* *< 10* years, 29% > 10 years, 83%	0.041* *< 10* weeks, 24% > 10 weeks, 62%	0.054 *< 100%*, 67% 100%, 30%
Feelings of depersonalization	0.27 F, 0% M, 25%	0.031 CG: 13% Non-CG, 7%	0.99 *≤* 5 years, 8% > 5 years, 12%	0.47 *< 10* years, 8% > 10 years, 17%	0.012* *< 10* weeks, 0% > 10 weeks, 23%	0.93 *< 100%*, 17% 100%, 9%

**%** of respondents who chose “Always” or “Often” shown in table

## Discussion

It has been well described in previous studies that physicians have been affected by occupational burnout leading to high rates of leaving the clinical work force or reducing their work hours [11–13]. The National Academy of Medicine recently launched a national plan for health workforce wellbeing that includes a recommendation for investing in measurement, assessment, strategies, and research to help reduce burnout and improve wellbeing. Wellbeing must be promoted at both an individual and an organizational level, and workplace environment can have a significant impact on the individual physician [8, 12]. Our study aimed to assess attending physician experience, attitudes, personal and work-related contributors to professional fulfillment (e.g., ability to teach learners on the team, feeling valued, symptoms of burnout) for those on General Medicine inpatient teaching services across the two major inpatient teaching sites for Wake Forest University School of Medicine. Academic physicians are role models for their learners. Burnout negatively impacts professionalism, patient safety, and patient satisfaction potentially affecting resident and medical student experience, which are the individuals who hold the future of our US healthcare system [14]. In our study, twenty-four (80%), seventeen (57%), and eleven (37%) attendings reported feeling either “often” or “sometimes” emotionally exhausted, reduced personal achievement, and depersonalization, respectfully. These findings are highly concerning because these as well-known contributors to reducing occupational wellness [8, 12, 13, 15].

Interestingly, most attendings in our study reported they were satisfied with their job. However, there are always opportunities to enhance the aspects of an academic attending’s job by improving areas they value highly, such as the ability to effectively teach residents and medical students as observed among junior academic hospitalists in prior studies [16]. In this study, teaching was one of the best parts of the job for early academic hospitalists, and it positively impacted job satisfaction [16]. Nonetheless, a common limitation noted to teaching learners was a lack of time [16]. As there has been a growing movement for Internal Medicine physicians to be solely inpatient-based or outpatient-based, it was interesting to note in our study that those Internal Medicine physicians working a 50/50 split of outpatient and inpatient clinical time felt that the type of admissions model and number of learners affected their ability to effectively teach. This is different than Berger et al.’s findings which reported that with a drip system the workload was more evenly distributed with more efficient use of physician resources without negatively affecting learner education in comparison to a bolus system [17]. It should be noted though that noon conference attendance was the metric used to assess learner education [17]. We also found that non-patient care obligations despite having administrative protected time may affect the ability to teach when on the wards. We can only postulate the factors contributing to this group’s barrier to effectively teach learners but suspect that the constant demands of administrative meetings and research obligations (to name a few) may hinder the ability to have a positive educational experience when on a General Medicine inpatient teaching service. Previous literature has shown that reducing resident-to-attending ratios on General Medicine wards can improve both parties’ experience, so this may be something to explore to help combat this issue [18]. Leadership may also consider innovative ways to distribute faculty protected time to make time on the teaching services more palatable. One change suggested is by dedicating specific time for each individual physician to devote a certain percentage of their work activities (20%) that is especially meaningful to them [19].

In terms of the workforce feeling valued, Simpkin et al., and West et al.’s findings suggest that a faculty’s sense of value within an organization is pivotal to their job satisfaction and overall wellbeing. When faculty feel recognized and appreciated for their contributions, it fosters a positive work environment and enhances morale. Recognition not only validates their efforts but also instills a sense of purpose, motivating them to perform at their best. This acknowledgment goes beyond monetary rewards, encompassing verbal appreciation, constructive feedback, and opportunities for professional growth [2, 8]. In our study, we found no significant findings in terms of attendings feeling less valued by hospital leadership, however there was a trend towards significance in attendings who are a primary caregiver to either a family member or friend and there were two specific comments in the qualitative question regarding having feelings of being undervalued by hospital leadership. Furthermore, those attendings with *≤* 5 years of experience supervising on a General Medicine inpatient teaching service felt less valued by internal medicine residency leadership. This finding is consistent with Zhuang et al., who found that those in a lower faculty rank (instructor, assistant/associate professor) when compared to the highest faculty rank (full professor) were less satisfied with their work [20].

Several limitations of the current study must be acknowledged. The overall response rate of 56% suggests a reasonably representative sample as evidenced by a recent meta-analysis estimating a mean response rate of 44.1% (95% confidence interval: 42.3–46.0%) for online survey studies [21]. However, the sample size did not allow for specific subgroup analysis in certain instances. Further, there was a significant difference between AHWFB and AHCMC in terms of the number of weeks on a General Medicine inpatient teaching service. AHCMC had significantly more attendings with > 10 weeks than AHWFB (*p* = 0.024) **(**Table 1**)**. We suspect this is likely due to a smaller cohort of attending physicians who supervise the General Medicine inpatient teaching services at AHCMC in comparison to AHWFB. Further, the survey questions used were not previously validated. However, the survey was created with input from an expert survey statistician, and we formulated specific questions regarding self-reported symptoms of burnout. Lastly, we do have hospital and internal medicine residency leadership at AHWFB and AHCMC who met the inclusion criteria to be included in this study. It is unclear if they participated in the study, but this also may have influenced the results of the study with underreporting in certain areas.

Future studies aimed at inpatient attendings who are primary caregivers, have less experience, and have more General Medicine inpatient clinical weeks with focus groups is warranted. The goal would be to ask them specific questions regarding factors that are contributing to their burnout and feelings of being devalued. Further, work-related factors such as flexible scheduling and providing opportunities for professional growth and coaching, may also be prudent to further measure impostor syndrome in these groups. Lastly, there is a potential opportunity to use our existing survey to across our own Advocate Health enterprise.

## Conclusions

In conclusion, the survey of attending physicians revealed insights into their experiences and perceptions while supervising General Medicine inpatient teaching services. Despite overall satisfaction with their roles, certain factors such as the admissions model, excessive learner saturation, and non-patient care obligations significantly impacted some attendings’ ability to effectively teach learners. Additionally, concerns about feeling valued by hospital and residency leadership, coupled with prevalent feelings of emotional exhaustion, depersonalization, and reduced personal achievement, underscore the need to explore targeted interventions to support attending physicians’ well-being and sense of value as an educator. Overall, these findings highlight the complexity of the attending physician role within academic medical centers and emphasize the importance of fostering environments conducive to both effective teaching and physician well-being. Our manuscript adds to previous literature [8, 12, 13, 15] that has shown that workplace environment contributes to occupational wellness and feeling valued. More studies need to be conducted, such as focus groups to ask attendings who are primary caregivers, have less experience, and have more General Medicine inpatient clinical weeks specific questions regarding factors contributing to burnout and feelings of being devalued factors. This may be helpful in determining the exact interventions that are needed to improve attending physician wellbeing, fulfillment and satisfaction. Lastly, there is also a potential future opportunity to assess wellbeing among attending physicians across the entire Advocate Health enterprise.

## Data Availability

Data is provided within the manuscript. If raw data is needed, these will be available from the corresponding author on reasonable request.

## Appendix

**Figure 1.** The purpose of the study is to evaluate faculty experience and perceptions on the **General Medicine Inpatient Teaching Services**. Your participation is completely **voluntary.** The study methods have been reviewed and approved by the Atrium Health Wake Forest Baptist IRB. All survey responses will be **anonymous** and collected by a neutral third-party investigator. **By completing this survey**,** you are consenting to participate in this study.** Any questions can be directed to any of the investigators (see below), and any concerns can be directed to the Wake Forest University School of Medicine IRB.

Thank you for participating.

Investigators:

Parag Chevli (pchevli@wakehealth.edu)

Chi Huang (chuang@wakehealth.edu)

Edward Ip (eip@wakehealth.edu)

Jacqueline Lippert (jdlipper@wakehealth.edu)

William Lippert (wlippert@wakehealth.edu)

Jessica McCutcheon (jessica.mccutcheon@atriumhealth.org)

Suma Menon (smenon@wakehealth.edu)

Christina Rinaldi (crinaldi@wakehealth.edu)

Gregory Russell (grussell@wakehealth.edu)

Kenneth Singhel (kenneth.singhel@atriumhealth.org)

1. To which gender do you most identify?
  - Female
  - Male
  - Non-binary
  - Transgender
  - Prefer not to say
2. Which of the following best describes you?
  - Asian or Pacific Islander
  - Black or African American
  - Hispanic or Latino
  - Multiracial or Biracial
  - Native American or Alaskan Native
  - White or Caucasian
  - A race/ethnicity not listed here
  - Prefer not to say
3. During the last academic year (July 1, 2022 – June 30, 2023), were you a primary caregiver to a family member (e.g., child; adult parent) or friend?
  - Yes
  - No
  - Prefer Not to Say
4. How many years have you been in practice (i.e., years as an attending physician)?
  - 0–2 years
  - 2–5 years
  - 6–10 years
  - 11–15 years
  - 15–20 years
  - > 20 years
5. How many years have you been a supervising attending on the **General Medicine Inpatient Teaching Services** at your CURRENT institution?
  - 0–2 years
  - 2–5 years
  - 6–10 years
  - 11–15 years
  - 15–20 years
  - > 20 years
6. How many years have you been a supervising attending on ANY **General Medicine Inpatient Teaching Services** (please include the number of years at your current institution PLUS any prior institutions)?
  - 0–2 years
  - 2–5 years
  - 6–10 years
  - 11–15 years
  - 15–20 years
  - > 20 years
7. Currently, in which setting do you primarily (e.g. >50% of your clinical time) work?
  - Inpatient
  - Outpatient
  - Both (if split 50% inpatient and 50% outpatient)
8. During the last academic year (July 1, 2022 – June 30, 2023), what was your **total** full-time equivalent (%)?
  - [Slider Rules]
9. Of your **total** full-time equivalent, what percentage of it is considered clinical time (i.e., percentage of working hours reserved for patient care duties)?
  - [Slider Rules]
10. Of your **total** full-time equivalent, what percentage of it is considered faculty protected time (i.e., percentage of working hours reserved for non-patient care duties, such as research, administration, education)?
  - [Slider Rules]

For all questions in this section, please answer them in reference to the last academic year (July 1, 2022 – June 30, 2023) while serving as a supervising attending on the **General Medicine Inpatient Teaching Services**?

11. Which site(s) did you work at?
  - Atrium Health Carolinas Medical Center only
  - Atrium Health High Point Medical Center only
  - Atrium Health Wake Forest Baptist Medical Center only
  - Both Atrium Health High Point Medical Center and Atrium Health Wake Forest Baptist Medical Center
12. How many weeks did you work as the supervising attending?
  - Less than 2 weeks
  - 2–4 weeks
  - 4–6 weeks
  - 6–8 weeks
  - 8–10 weeks
  - More than 10 weeks
13. How satisfied were you with your overall experience?
  - Very Satisfied
  - Satisfied
  - Dissatisfied
  - Very Dissatisfied
  - Prefer Not to Say
14. How often did you feel that you had adequate time to teach the residents and medical students on rounds?
  - Always
  - Often
  - Sometimes
  - Rarely
  - Never
15. How often did you feel that you had adequate time for dedicated teaching sessions (e.g., chalk talks) to teach the residents and medical students?
  - Always
  - Often
  - Sometimes
  - Rarely
  - Never
16. How often did you feel that the number of residents and medical students affected your ability to effectively teach them?
  - Always
  - Often
  - Sometimes
  - Rarely
  - Never
17. How often did you feel that the patient census affected your ability to effectively teach the residents and medical students?
  - Always
  - Often
  - Sometimes
  - Rarely
  - Never
18. How often did you feel that the patient admission model (e.g., drip vs. bolus system) affected your availability to effectively teach residents and medical students? For clarification: A bolus system is used at Atrium Health Carolinas Medical Center; a drip system is used at Atrium Health High Point Medical Center; and a drip system is used at Atrium Health Wake Forest Baptist Medical Center.
  - Always
  - Often
  - Sometimes
  - Rarely
  - Never
19. How often did you feel that non-patient care obligations (e.g., administrative meetings; research) affected your availability to effectively teach residents and medical students?
  - Always
  - Often
  - Sometimes
  - Rarely
  - Never
20. How often did you feel that you had adequate time to provide feedback to residents and medical students?
  - Always
  - Often
  - Sometimes
  - Rarely
  - Never
21. How often did you feel that the length of your on-service block (e.g., 7-days in a row, or 14-days in a row) affected your availability to effectively teach the residents and medical students?
  - Always
  - Often
  - Sometimes
  - Rarely
  - Never
22. How often did you feel that current system initiatives/quality metrics* (e.g., prioritizing early discharges) affected your availability to effectively teach residents and medical students?
  - Always
  - Often
  - Sometimes
  - Rarely
  - Never
23. How often did you feel valued by hospital leadership?
  - Always
  - Often
  - Sometimes
  - Rarely
  - Never
24. How often did you feel valued by the internal medicine residency leadership?
  - Always
  - Often
  - Sometimes
  - Rarely
  - Never
25. How often did you feel valued by the residents and medical students?
  - Always
  - Often
  - Sometimes
  - Rarely
  - Never
26. How often did you feel that non-patient care obligations (e.g., administrative meetings; research) affected your work-life balance?
  - Always
  - Often
  - Sometimes
  - Rarely
  - Never
27. How often did you feel emotionally exhausted due to work?
  - Always
  - Often
  - Sometimes
  - Rarely
  - Never
28. How often did you have feelings of depersonalization*?
  - Always
  - Often
  - Sometimes
  - Rarely
  - Never
29. How often did you have feelings of reduced personal accomplishment?
  - Always
  - Often
  - Sometimes
  - Rarely
  - Never
30. Feel free to share any other comments or suggestions on improving your experience while serving as a supervising attending for the **General Medicine Inpatient Teaching Services**:
  - [Open-ended Answer]

*Please note that for question #22, the current system initiatives / quality metrics included:

- For AHWFB:
  - Discharges by 10am
  - Hospitalist at Home Referral Rate
  - Discharge Summary Completion Rate within 48 h

- For AHCMC:
  - 30-day readmissions
  - Hierarchical condition category (HCC) capture

*Please note that for question #27, the definition of depersonalization should be from the Maslach Burnout Inventory, which defines depersonalization as a component of burnout, specifically referring to the development of negative or cynical attitudes and feelings towards one’s work or the recipients of one’s care. It reflects a sense of emotional withdrawal or detachment from the individuals being served, leading to impersonal interactions and decreased empathy. (Source: Maslach, Christina & Jackson, Susan & Leiter, Michael. (1997). The Maslach Burnout Inventory Manual.)

**Figure 2.**

Dear Colleague:

Researchers at Atrium Health Wake Forest Baptist Medical Center and Atrium Health Carolinas Medical Center are inviting you to take part in a survey about **faculty experience** on the **General Medicine Inpatient Teaching Services.**

There are no known risks to participating in this study other than a potential breach of confidentiality. However, we will make every effort to minimize this risk. Your responses to the survey will be completely **anonymous** and **confidential** to the extent allowed by law. Further, a neutral-third party who has absolutely no role in deciding Atrium Health Wake Forest Baptist Medical Center, Atrium Health High Point Medical Center, and Atrium Health Carolinas Medical Center attending roles, responsibilities, or remuneration will analyze your responses.

If you have questions about the study, please feel free to contact the Principal Investigator, William Lippert (wlippert@wakehealth.edu; office: (336) 713–7067). If you have complaints, suggestions, or questions about your rights as a research volunteer, contact the staff at Wake Forest University School of Medicine IRB at (336) 716–4542. The IRB number is IRB00095925.

Here is a **link** to the RedCap survey: [Individualized RedCap link here]

By completing this survey, you are consenting to participate in this study. Please complete by **August 12**,** 2023 at 11:59pm**.

Thank you in advance for your assistance with this important project.

Sincerely,

Parag Chevli (pchevli@wakehealth.edu)

Chi Huang (chuang@wakehealth.edu)

Edward Ip (eip@wakehealth.edu)

Jacqueline Lippert (jdlipper@wakehealth.edu)

William Lippert (wlippert@wakehealth.edu)

Jessica McCutcheon (jessica.mccutcheon@atriumhealth.org)

Suma Menon (smenon@wakehealth.edu)

Christina Rinaldi (crinaldi@wakehealth.edu)

Gregory Russell (grussell@wakehealth.edu)

Kenneth Singhel (kenneth.singhel@atriumhealth.org)

## Abbreviations

AHCMC: Atrium Health Carolinas Medical Center
AHWFB: Atrium Health Wake Forest Baptist
US: United States
